# The roles of microRNAs and siRNAs in mammalian spermatogenesis

**DOI:** 10.1242/dev.136721

**Published:** 2016-09-01

**Authors:** Stephanie Hilz, Andrew J. Modzelewski, Paula E. Cohen, Andrew Grimson

**Affiliations:** 1 Department of Molecular Biology and Genetics, Cornell University, Ithaca, NY 14853, USA; 2 Department of Biomedical Sciences, Cornell University, Ithaca, NY 14853, USA; 3 Department of Molecular and Cell Biology, University of California Berkeley, Berkeley, CA 94720, USA

**Keywords:** AGO, Germ line, Mammalian spermatogenesis, miRNA, MicroRNA, siRNA

## Abstract

MicroRNAs and siRNAs, both of which are AGO-bound small RNAs, are essential for mammalian spermatogenesis. Although their precise germline roles remain largely uncharacterized, recent discoveries suggest that they function in mechanisms beyond microRNA-mediated post-transcriptional control, playing roles in DNA repair and transcriptional regulation within the nucleus. Here, we discuss the latest findings regarding roles for AGO proteins and their associated small RNAs in the male germline. We integrate genetic, clinical and genomics data, and draw upon findings from non-mammalian models, to examine potential roles for AGO-bound small RNAs during spermatogenesis. Finally, we evaluate the emerging and differing roles for AGOs and AGO-bound small RNAs in the male and female germlines, suggesting potential reasons for these sexual dimorphisms.

## Abstract (precis)

**Summary:** This Review article summarizes the latest findings regarding roles for AGO proteins and their associated small RNAs in the male germline, with a particular focus on spermatogenesis.

## Introduction

**Fig. 1.**
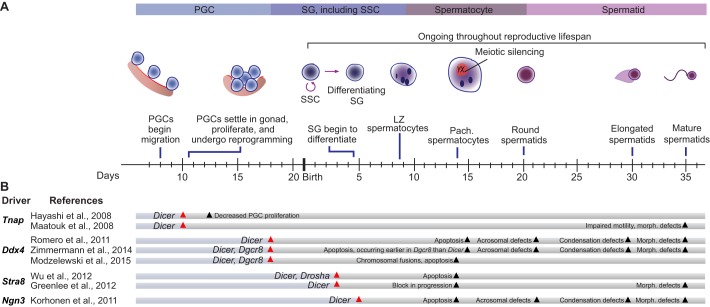
**Spermatogenesis in mice and the effects of *Dgcr8*, *Drosha* and *Dicer* male germline knockout.** (A) In male mice, PGCs begin to migrate to the gonad at embryonic day 8 (E8). By E10.5, they settle in the gonad and begin a program of proliferation and reprogramming, eventually becoming spermatogonia (SG). Around postnatal day 4.5 (P4.5), a subset of spermatogonia further differentiate and enter meiosis at P8, becoming spermatocytes. Of the spermatogonia that remain, some serve as SSCs, which replenish the spermatogonial pool for future waves of spermatogenesis. Following completion of meiosis at P20, germ cells (now called round spermatids) undergo morphological changes, including chromatin condensation and cellular elongation, ultimately forming mature spermatids. Following this initial wave of spermatogenesis, the process reoccurs throughout the organism's reproductive lifespan. (B) The conditional knockout of genes encoding small RNA biogenesis factors (*Dgcr8*, *Drosha* or *Dicer*) reveals essential roles for AGO-bound small RNAS during spermatogenesis. Summarized here are studies in which *Dgcr8*, *Drosha* or *Dicer* were conditionally disrupted from the male mouse germline. The promoter driving Cre-mediated disruption in each case is indicated [*Tnap* (*Alpl*); *Ngn3* (*Neurog3*)], together with the approximate time of Cre activation (red arrowhead) and the time point at which a phenotype was observed (black arrowhead). Timescale indicates embryonic days up until birth, then postnatal days. LZ, leptotene/zygotene; Morph., morphological; Pach., pachytene.

The process of spermatogenesis involves complex, dynamic patterns of gene regulation and chromatin rearrangement. In early embryogenesis (Fig. 1A), mammalian primordial germ cells (PGCs) are specified as germ cells and begin a program of rapid proliferation, reprogramming, and transposon silencing (Saitou et al., 2012), ultimately becoming spermatogonia. At sexual maturity, a subset of spermatogonia enter meiosis, whereas others remain, serving as spermatogonial stem cells (SSCs) that replenish the spermatogonial pool (de Rooij, 2001). In waves continuing throughout the reproductive lifespan, subsets of the spermatogonial pool enter meiosis (de Rooij, 2001), becoming spermatocytes. During meiosis, homologous chromosomes pair to facilitate recombination and crossing over (Hunter, 2015), which is dependent on the induction of double-strand breaks (Borde and de Massy, 2013). Unpaired DNA is transcriptionally silenced during the pachytene stage of meiosis; this silencing, which we refer to as meiotic silencing, includes silencing of the unpaired X and Y chromosomes across almost their entire length (Turner, 2015). After completion of meiosis, spermatids densely compact their chromatin, yet retain expression of genes required for chromatin repackaging and cellular morphogenesis (Braun, 1998).

Recent studies have revealed that small RNAs play essential roles during many of the events that occur during spermatogenesis. Small RNAs are short non-coding RNAs that function by guiding a crucial co-factor – an AGO or PIWI protein of the Argonaute family – to target RNAs. Through this ability to guide regulatory complexes to RNA transcripts in a sequence-specific manner, small RNAs comprise an elegant system of gene regulation. Since their discovery (Lee et al., 1994; Wightman et al., 1994), it has become clear that they impact virtually every regulatory pathway in mammals (Kim et al., 2009). Thus, it is perhaps no surprise that small RNAs play crucial roles in the mammalian male germline, which undergoes dramatic epigenetic reprogramming events, complex transcriptional regulation, and structural metamorphosis to complete spermatogenesis.

In mammals, and most animals, there are three classes of small RNAs – microRNAs (miRNAs), endogenous small interfering RNAs (siRNAs) and PIWI-interacting RNAs (piRNAs) (Kim et al., 2009) – all three of which are present in the male germline. Though scarce in somatic cells, piRNAs are abundant in male germ cells and comprise the majority of small RNAs present during certain stages of germ cell development. The PIWI proteins that associate with piRNAs, of which there are three in mice, are also expressed in the male germline and are essential for fertility (Carmell et al., 2007; Deng and Lin, 2002; Kuramochi-Miyagawa et al., 2004), underscoring the essential role of the piRNA pathway in spermatogenesis (reviewed by Fu and Wang, 2014). The appearance of piRNAs occurs in two distinct waves during spermatogenesis: one in PGCs, producing what are known as pre-pachytene piRNAs, and the other during the pachytene stage of meiotic prophase I, producing pachytene piRNAs (Meikar et al., 2011). An essential function of pre-pachytene piRNAs is to repress transposon activity by directing the *de novo* methylation of transposon-encoding genes, thus protecting the germline genome from detrimental transposon accumulation (De Fazio et al., 2011; Kuramochi-Miyagawa et al., 2008; Siomi et al., 2011). Although a small proportion of pachytene piRNAs also function to repress transposons, in this case through a post-transcriptional mechanism that involves direct cleavage of the target transposon transcript (Di Giacomo et al., 2013; Reuter et al., 2011), roles for the majority of pachytene piRNAs remain to be defined; one intriguing possible function is the elimination of mRNAs during spermatid formation (Gou et al., 2014).

Beyond their piRNA populations, male germ cells also express a developmentally dynamic landscape of miRNAs and siRNAs (Hayashi et al., 2008; Song et al., 2011). Compared with our understanding of piRNAs, our comprehension of germline miRNAs and siRNAs is in its infancy. Nonetheless, recent studies are beginning to reveal essential roles for these RNAs during spermatogenesis. Uncovering the potential roles that they play in germ cells will deepen our understanding of spermatogenesis and holds the potential to reveal novel mechanisms of small RNA-mediated regulation in mammals.

Here, we review the emerging roles of AGO-bound small RNAs, which include miRNAs and siRNAs, during spermatogenesis. We begin by providing an overview of Argonaute proteins, focusing on the AGO subclade and their associated small RNAs. We highlight the mechanisms by which these AGO-bound small RNAs are generated, and how they function to regulate their targets. We next summarize what is known about the identities of miRNAs required for spermatogenesis, and we also describe the characterization of germline siRNAs, which currently are not thought to play an essential role in male germ cell development. We then discuss recent studies that have identified roles for AGO-bound small RNAs in the male germline, integrating recent findings in mammalian somatic cells with those in the germline to speculate on the mechanisms by which small RNAs might function, including novel nuclear roles in heterochromatin formation and DNA damage repair. Finally, we discuss the differing roles for AGO-bound RNAs in the male and female germlines, suggesting potential reasons for why this sexual dimorphism might exist.

## An introduction to Argonaute proteins and AGO-associated small RNAs

The Argonaute family comprises a group of deeply conserved proteins (Höck and Meister, 2008) found in almost all eukaryotes. Although some organisms, such as *Schizosaccharomyces* *pombe*, possess only a single Argonaute, many have multiple Argonautes, which are often functionally specialized; in *Drosophila* *melanogaster* there are five, *Homo* *sapiens* eight, *Arabidopsis* *thaliana* ten, and *Caenorhabditis* *elegans* twenty seven (Höck and Meister, 2008). In animals, the Argonaute family has diverged into two clades: AGO and PIWI (Tolia and Joshua-Tor, 2007). The biogenesis of small RNAs, and whether they associate with an AGO or a PIWI protein, are the major characteristics that distinguish the different classes of small RNAs. For example, piRNAs, which are ∼26-32 nucleotides (nts) in length, associate exclusively with PIWI proteins (Aravin et al., 2006; Girard et al., 2006). By contrast, miRNAs, which are ∼19-23 nts long and are, in general, the best-characterized small RNA class, associate exclusively with AGO proteins (Kim et al., 2009). Although miRNAs are found in all cell types, individual miRNAs are typically expressed in a tissue-specific manner (Lagos-Quintana et al., 2002). Mature miRNAs originate from larger hairpin-forming transcripts, which are recognized by the RNA-binding protein DGCR8 and processed by the nuclease DROSHA, releasing the hairpin from the primary transcript (Denli et al., 2004; Faller et al., 2010; Gregory et al., 2004; Landthaler et al., 2005). The hairpin precursor miRNA is then further processed by the nuclease DICER (DICER1), producing the mature miRNA, which is loaded onto an AGO protein, generating the effector complex (Kim et al., 2009; Lund and Dahlberg, 2006). Mammalian genomes contain hundreds of different miRNAs, many of which are deeply conserved (Griffiths-Jones et al., 2006; Kozomara and Griffiths-Jones, 2014; Lagos-Quintana et al., 2001). siRNAs are ∼19-23 nts in length and, like miRNAs, associate exclusively with AGO proteins (Kim et al., 2009); however, their biogenesis distinguishes them from miRNAs. Any transcript capable of forming double-stranded structures, either inter- or intramolecularly, has the potential to be processed into an siRNA. Unlike miRNA biogenesis, siRNA biogenesis requires neither DGCR8 nor DROSHA; siRNAs are processed solely by the enzyme DICER (Kim et al., 2009; Tam et al., 2008; Watanabe et al., 2008). siRNAs have been detected in mammalian embryonic stem cells (ESCs), oocytes and spermatocytes (Babiarz et al., 2008; Song et al., 2011; Tam et al., 2008; Watanabe et al., 2008). Because any double-stranded RNA (dsRNA) can generate siRNAs, which are only rarely conserved in sequence, a major challenge in the small RNA field is to differentiate between siRNAs possessing functional relevance and those that do not.

Upon AGO binding, both miRNAs and siRNAs guide the AGO complex to target RNAs containing sequences that are complementary to those of the small RNA. The subsequent association of an AGO protein with a target mRNA in mammals leads either to transcript cleavage or to the recruitment of additional factors that promote translational repression and destabilization of the targeted transcript (Hu and Coller, 2012). Target cleavage occurs only when extensive complementarity exists between the small RNA and target; we refer to this mechanism of gene regulation as ‘cleavage dependent’. Cleavage is also contingent on the identity of the AGO protein; in mammals, there are four AGO proteins (AGO1, 2, 3 and 4), and only AGO2 possesses the ability to cleave mRNAs (Liu et al., 2004; Wang et al., 2009). Almost every characterized mammalian miRNA-target interaction involves only a small region of complementarity, comprising pairing of 6-7 nts at the 5′ end of the miRNA, known as the ‘seed’ region, and a complimentary target site located in the mRNA 3′ untranslated region (3′ UTR); for many miRNAs, such target sites tend to be preferentially conserved (Bartel, 2009). Therefore, regardless of the AGO with which a particular miRNA associates, accelerated transcript decay and translational repression, rather than cleavage, are the dominant modes of action of mammalian miRNAs (Haley and Zamore, 2004; Martinez and Tuschl, 2004). Because they target near-identical sets of mRNAs, miRNAs with common seed sequences are grouped into families; indeed, many mammalian miRNAs exist as families, with individual members often functioning as redundant family members (Alvarez-Saavedra and Horvitz, 2010; Linsley et al., 2007).

Mammalian siRNA-target relationships are, by contrast, poorly characterized, although in other model systems (e.g. flies, yeast), they often involve extensive base-pairing and thus can lead to transcript cleavage (Buker et al., 2007; Czech et al., 2008; Nakanishi et al., 2012; Piatek and Werner, 2014). Cleavage-independent modes of siRNA-mediated regulation also exist (Doench et al., 2003; Saxena et al., 2003). Furthermore, beyond targeting mRNAs for post-transcriptional repression, which occurs in the cytoplasm, recent findings have suggested that mammalian AGO-bound small RNAs regulate gene expression in the nucleus, although their mechanism(s) of action in this context remain largely uncharacterized (Carissimi et al., 2015; Francia et al., 2012; Gao et al., 2014; Skourti-Stathaki et al., 2015).

## AGO-bound small RNAs are required for spermatogenesis

Conditional knockout (cKO) mouse models in which the small RNA biogenesis factors DGCR8, DROSHA or DICER were disrupted specifically in the male germline were the foundational experiments that revealed essential roles for AGO-bound small RNAs in spermatogenesis (Fig. 1B). The phenotypes of *Dgcr8*, *Drosha* and *Dicer* germline cKOs are largely congruent, and include infertility (or subfertility), decreased sperm count and disrupted sperm morphology. Although DGCR8, DROSHA and DICER are all small RNA biogenesis factors, they also possess additional, non-overlapping roles in the cell (Macias et al., 2012; White et al., 2014; Wu et al., 2000). However, the overall consistency of phenotypes among all three conditional knockouts indicates that absence of small RNAs, and miRNAs in particular, is causative, rather than a loss of the varied ancillary functions of DGCR8, DROSHA and DICER*.* These cKO studies, which employed a variety of promoters to drive germline-specific gene disruption, also revealed that the timing of Cre-mediated disruption of *Dgcr8*, *Drosha* or *Dicer* affects the identity and severity of spermatogenic phenotypes (Fig. 1B). For example, the early disruption of *Dicer* at embryonic day (E) 10 impedes PGC proliferation (Hayashi et al., 2008) and spermatid elongation (Maatouk et al., 2008). By contrast, the disruption of *Dicer*, *Dgcr8* or *Drosha* at later time points, at E18 or postnatal day (P) 3, results in defective progression from the leptotene/zygotene stage of meiotic prophase I to the pachytene stage (Greenlee et al., 2012), ultimately resulting in the elimination of spermatocytes at pachytene (Modzelewski et al., 2015; Romero et al., 2011; Wu et al., 2012; Zimmermann et al., 2014). Finally, *Dicer* disruption at P5 results predominantly in post-meiotic phenotypes, with defects in spermatid condensation and elongation (Korhonen et al., 2011). Together, these studies demonstrate that AGO-bound small RNAs are not only essential for spermatogenesis, but are likely to participate in multiple stages of spermatogenesis including, at least, PGC proliferation, meiotic prophase I, and spermatid elongation.

Beyond revealing that AGO-bound small RNAs are essential for multiple aspects of spermatogenesis, cKO studies offer valuable insights into which class of AGO-bound small RNAs – miRNAs or siRNAs – underlie a given phenotype. Defects common to *Dicer*, *Dgcr8* and *Drosha* cKOs imply that disruption of miRNAs is causative, whereas a defect specific to *Dicer* cKO animals suggests that siRNAs are crucial for the process. Indeed, comparative studies revealed similar defects in *Dicer* and *Dgcr8*, as well as *Dicer* and *Drosha*, cKOs during meiotic prophase I and spermatid elongation, thus implicating miRNAs, and not siRNAs, as factors essential to both processes (Fig. 1B). However, the phenotypic severities of *Dgcr8*, *Drosha* and *Dicer* cKOs are not identical; comparisons of *Dgcr8* and *Dicer* cKOs reveal more severe phenotypes in *Dicer* cKOs (Modzelewski et al., 2015; Zimmermann et al., 2014), suggesting that siRNAs also play a role in the male germline, with such roles potentially masked by the concurrent loss of miRNAs in *Dicer* cKOs. Although intriguing, such discrepancies in phenotypic severity might derive from technical limitations, such as differences in the stability of residual proteins after gene deletion, or to secondary functions of DGCR8, DROSHA and DICER independent of miRNA and siRNA biogenesis (Macias et al., 2012; White et al., 2014; Wu et al., 2000). Indeed, one study comparing *Dicer* and *Drosha* cKOs found more severe defects in the absence of *Drosha* (Wu et al., 2012). Taken together, these studies demonstrate essential roles for miRNAs in spermatogenesis, but leave the role of siRNAs unsubstantiated.

A major limitation of the cKO studies described above is that disruption of small RNA biogenesis affects multiple small RNAs and pathways simultaneously. Thus, although the analyses of cKO models have established the importance of AGO-bound small RNAs in PGC proliferation, meiosis, and spermatid condensation, they offer no insight into the identities of the specific small RNAs underlying the phenotypes, nor do they reveal the functional roles of such small RNAs. Disruption of even a single miRNA typically alters the regulation of many dozens of direct targets, which themselves can regulate the expression of additional genes (indirect targets), resulting in a network of downstream effects. The difficulties in identifying the misregulated processes are reflected by the wide variety of explanations for infertility in miRNA-deficient germlines, including upregulation of gene expression from the X and Y chromosomes (Greenlee et al., 2012; Wu et al., 2012), an increase in SINE expression (Romero et al., 2011), chromosome instability and alterations in the DNA damage pathway (Modzelewski et al., 2015), and an upregulation of centromeric repeat transcripts (Korhonen et al., 2011). Therefore, and as we discuss below, additional approaches have been necessary to determine which specific miRNAs are important during spermatogenesis, what specific processes they affect and whether other types of AGO-bound small RNAs also play a role in spermatogenesis.

## The identification of specific miRNAs with roles in the male germline

Multiple approaches have been employed to tease out the identities of specific miRNAs that, when disrupted, lead to the germ cell phenotypes observed in *Dgcr8*, *Drosha* and *Dicer* cKOs. These studies have highlighted essential roles for the miR-34 family in spermatogenesis in mice, and suggest that members of the miR-17-92 cluster (Mirc1) might also perform important functions.

The miR-34 family (miRNAs with the seed sequence GGCAGUG) had previously been postulated to have a role in the testis, largely owing to its preferential and strong expression in the male germline (Bao et al., 2012; Wu et al., 2014). Expression of this family sharply increases with the onset of meiosis (Bao et al., 2012; Bouhallier et al., 2010; Liang et al., 2012), and family members are also expressed in SSCs (Niu et al., 2011) and spermatozoa (García-López et al., 2015). The miR-34 family comprises six miRNAs distributed among three chromosomes (Griffiths-Jones et al., 2006; Kozomara and Griffiths-Jones, 2014). Early studies disrupting only a subset of these loci did not result in infertility or detectable disruptions in spermatogenesis (Bao et al., 2012; Concepcion et al., 2012). Importantly, partial deletion of the family led to upregulated expression of remaining family members, suggesting compensation among family members (Bao et al., 2012). Indeed, it was necessary to disrupt five of the six miRNA-encoding loci to uncover the essential germline role of the miR-34 family (Comazzetto et al., 2014; Song et al., 2014; Wu et al., 2014). These miR-34 family knockout mice are infertile; moreover, they produce few mature sperm, and those they do produce are abnormal (Comazzetto et al., 2014; Song et al., 2014; Wu et al., 2014). Interestingly, germ cell loss in miR-34 family knockouts occurs at two distinct phases: during pachytene (Comazzetto et al., 2014) and later during spermatid elongation (Comazzetto et al., 2014; Song et al., 2014; Wu et al., 2014). These patterns of spermatocyte and spermatid loss are strikingly similar to those observed in *Dgcr8*, *Drosha* and *Dicer* cKOs, suggesting that loss of the miR-34 family in these cKO animals probably contributes to many of the observed defects. Notably, it was observed that PGCs appear normal in miR-34 family knockout mice (Comazzetto et al., 2014). This result suggests that the loss of this miRNA family does not contribute to the proliferation defect observed in *Dicer* cKO PGCs, implicating roles for additional AGO-bound small RNAs in spermatogenesis.

Knockout mouse models also provided intriguing evidence to suggest that the miR-17-92 cluster – a locus containing miRNAs belonging to four different families (miR-17, -18, -19 and -25) – might contribute to early spermatogonial differentiation. Germline conditional loss of the miR-17-92 cluster, driven by *Ddx4* Cre, resulted in reduced testis size and weight, with many tubules containing only Sertoli cells, although the mice are fertile (Tong et al., 2012). However, similar to the miR-34 family, several miRNAs in the cluster have paralogs elsewhere in the genome, all of which are upregulated in miR-17-92 cluster knockout mice, potentially masking a stronger phenotype (Tong et al., 2012). Taken together, it seems likely that one or more of the miRNAs within the miR-17-92 cluster play major roles in the male germline. Utilizing mouse knockout strategies focused on miRNA families, as opposed to individual miRNAs or clusters, will be required to define potential roles in spermatogenesis for members of the miR-17-92 cluster, as well as for the large proportion of other miRNAs that belong to multi-copy families.

**Fig. 2.**
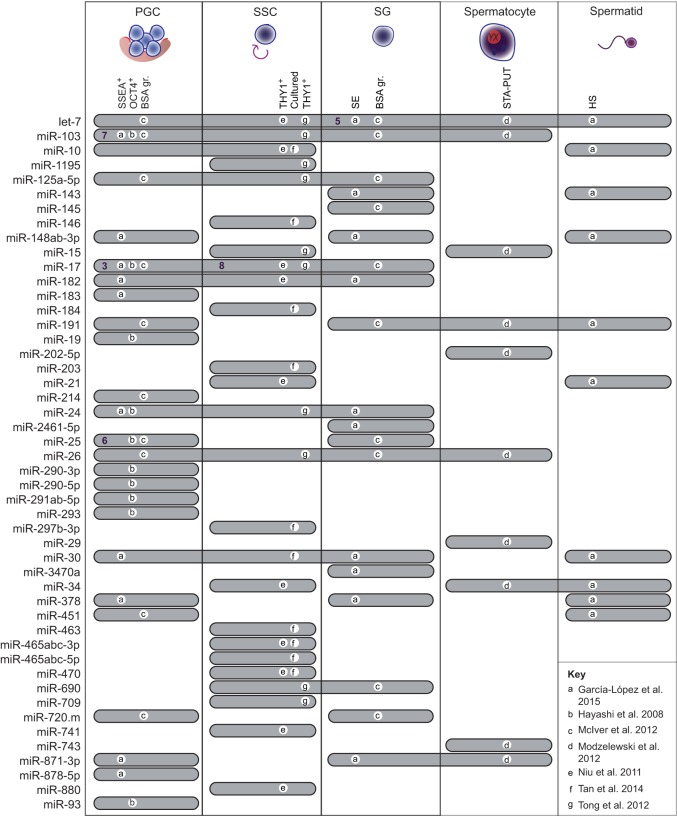
**Highly expressed miRNA families in purified mouse spermatogenic cells.** Cells were purified using different techniques (gr., gradient; HS, hyposmotic shock; SE, somatic elimination; STA-PUT, a velocity sedimentation technique for separation) or markers [SSEA (FUT4); OCT4 (POU5F1)]. miRNA families (far left) are referenced by the first well-known member of each family. Gray shaded bars indicate that a miRNA family is among the top ten most highly expressed families in at least one study and in at least one of the indicated germline cell types. The relevant references are indicated by the letters a-g; see key in bottom right. The average rank expression is shown (bold numbers) for those miRNAs for which the average rank expression across multiple studies was within the top ten.

Analysis of the expression patterns of miRNAs, together with clinical studies investigating expression changes in infertile men, have identified many other miRNAs with potential roles in spermatogenesis. Although high expression of a miRNA does not necessarily imply functionality, only highly expressed miRNAs are likely to have an impact on target expression (Bosson et al., 2014; Mukherji et al., 2011). The most highly expressed miRNA families at different stages of spermatogenesis have been identified using microarrays, qPCR, and small RNA sequencing (Fig. 2). One difficulty in the interpretation of these data is that relative miRNA quantification varies between different types of assays. Moreover, miRNA profiles are highly sensitive to the purity of isolated cells. The impact of these technical considerations is evident when one compares the most abundant miRNA families identified in different studies, and highlights the benefit of integrating data from multiple studies when searching for new candidates with potentially interesting functions (Fig. 2). Notably, additional studies have found correlations between infertility and altered expression of specific miRNA families in spermatozoa or seminal fluid, including a decrease in miR-34 family member levels (Abu-Halima et al., 2013, 2014; Wang et al., 2011). However, it is not possible to know whether altered levels of miRNAs are causative factors contributing to infertility or downstream consequences of the underlying defect(s). Despite such limitations, these studies, combined with those characterizing highly or differentially expressed miRNAs, have identified many miRNA families of interest and provided numerous candidates, with miR-103 and miR-17 among the most promising, owing to their reproducible high expression in early germ cells (Fig. 2), for further investigation using knockout mice.

Together, miRNA knockout mouse studies have demonstrated that the miR-34 family is required for spermatogenesis. Given that multiple miRNA families are dynamically expressed at various stages during spermatogenesis, it is likely that many other miRNA families are also essential. There is strong evidence that at least one of these miRNA families belongs to the miR-17-92 cluster. Finally, miRNA profiling experiments have identified many miRNAs, including miR-103 and miR-17, as being highly expressed during certain stages of gametogenesis, indicating that they might also play an important role in the regulation of male germ cell gene expression.

## The identification of siRNAs in the male germline

As for miRNAs, various approaches have been used to identify siRNAs within the male germline. Notably, small RNA sequencing has provided comprehensive small RNA expression profiles of germ cells at various stages of spermatogenesis and has identified dozens of presumptive siRNAs in male germ cells (Song et al., 2011; Tan et al., 2014). A recent study, for example, found that siRNAs are highly expressed in SSCs (Tan et al., 2014), congruent with previous discoveries of siRNAs in ESCs (Babiarz et al., 2008). An earlier study characterized siRNAs in the developing testis, finding that about half of the siRNA population is expressed during specific stages of spermatogenesis, including many upon meiotic entry, whereas the remaining half is expressed more ubiquitously throughout spermatogenesis (Song et al., 2011). In the female germline, the majority of siRNAs are derived from transposable elements and help to silence them (Babiarz et al., 2008; Watanabe et al., 2008); in the male germline, however, siRNAs are typically derived from non-transposon loci distributed across the genome, with no clear functions yet ascribed (Song et al., 2011). Double-stranded precursors could be identified for some, but not all, male-specific siRNAs, and the presence of many was dependent on *Dicer* but not *Drosha* (Song et al., 2011), giving credence to their classification as siRNAs. Although their low expression levels in male germ cells have cast doubts on their functional significance, it should be noted that siRNAs expressed at very low levels in other cell types still appear to be able to impact chromatin dynamics (Carissimi et al., 2015). It is also worth noting, however, that with the increasing power of sequencing, ever increasing numbers of low-abundance siRNAs will continue to be described, challenging the field to develop new ways to distinguish which, if any, of these newly discovered small RNAs play meaningful biological roles.

In contrast to miRNAs, siRNAs haven been difficult to target via knockout approaches owing to their dispersion across many loci in the genome and difficulty in confidently identifying which of these disparate loci are redundant. To date, the most straightforward method is comparative analysis of phenotypes between *Dicer* and *Dgcr8*, as well as *Dicer* and *Drosha*, cKOs. Given the known roles of low-abundance siRNAs in regulating chromatin dynamics (Carissimi et al., 2015), future comparisons of chromatin markers between *Dgcr8* or *Drosha* and *Dicer* cKOs would be especially useful for revealing whether siRNAs might be playing an important regulatory role in male gametogenesis.

## The functions of AGO-bound small RNAs during spermatogenesis

**Fig. 3.**
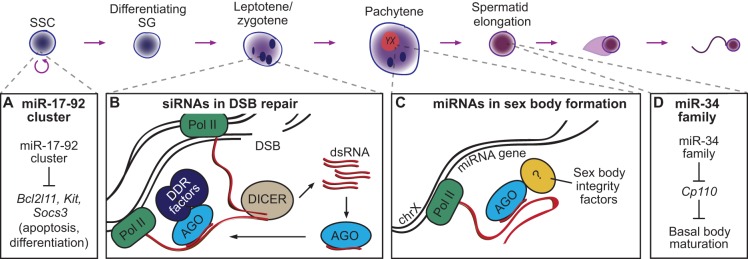
**Potential roles for AGO-bound small RNAs during spermatogenesis*.*** (A) There is evidence to suggest that the miR-17-92 miRNA cluster is important for maintenance of spermatogonia in an undifferentiated state. (B) Observations in somatic cells suggest that AGO-bound small RNAs in the nucleus help repair DSBs, which are introduced to facilitate synapsis during leptotene. In such a model, divergent transcription from sites of DSBs produces dsRNA, which is cleaved by DICER and loaded onto AGO proteins. AGO proteins can then be guided back to these sites via base pairing between DSB-associated small RNAs and precursor transcripts, where they can recruit DNA damage response (DDR) factors. (C) During pachytene, AGO-bound small RNAs are important for proper sex body formation. In the model shown here, miRNA-encoding genes that escape silencing on the X chromosome produce miRNAs that are loaded onto AGO proteins. AGO proteins are then guided back to their locus of origin via base pairing between miRNAs and precursor transcripts. AGO proteins recruit unknown sex body integrity factors to form the sex body's tertiary chromatin structure, in which miRNA loci are localized to the periphery and escape silencing. (D) The miR-34 family is essential during pachytene, and also for spermatid elongation, for which it is hypothesized to play a role in basal body maturation and hence spermatid flagellum formation.

The simplest explanation for how AGO-bound small RNAs function during spermatogenesis is that they behave as canonical miRNAs, acting as post-transcriptional regulators of specific target mRNAs in the cytoplasm. However, it is becoming evident that mammalian AGO-bound small RNAs can also function in the nucleus to influence chromatin dynamics. For example, AGO proteins are found within the mammalian nucleus (Gagnon et al., 2014; Robb et al., 2005; Rüdel et al., 2008) and associate with chromatin (Ameyar-Zazoua et al., 2012; Benhamed et al., 2012; Huang et al., 2013). Moreover, animal genomes, including those of mammals, typically encode multiple AGO proteins, which in non-mammalian animals are often functionally specialized, some possessing nuclear roles (Ashe et al., 2012; Huang and Li, 2014). Intriguingly, deletion of AGO2, the sole cleavage-competent mammalian AGO protein, in the mouse germline does not impair spermatogenesis (Hayashi et al., 2008), suggesting that nuclear AGO proteins cannot rely upon target cleavage. In addition, although AGO-like proteins in bacteria interact directly with DNA, mammalian AGOs are thought not to possess this ability (Salomon et al., 2015). Although there are many possible ways by which AGO proteins could function in the mammalian germline nucleus, evidence from non-mammalian organisms implicates them in several distinct nuclear processes during spermatogenesis, including heterochromatin formation, transcriptional gene silencing and DNA damage repair (Fig. 3).

### Cytoplasmic functions for AGO-bound small RNAs

As mentioned above, the miR-34 family plays an essential role in spermatogenesis, and there is strong evidence that it does so by promoting flagellum formation, mediated, at least in part, through repression of the *Cp110* transcript (Fig. 3), which encodes a protein that inhibits basal body maturation (Song et al., 2014). However, although *Cp110* regulation could explain why many spermatids from miR-34 family mutants are blocked during elongation, it does not explain why additional miR-34 family-deficient germ cells also arrest well before this stage, in meiosis. During meiosis, the miR-34 family might instead help regulate the elimination of defective spermatocytes by apoptosis, as suggested by a study showing that the miR-34 family targets *Atf1*, which encodes a protein implicated in germ cell apoptosis (Liang et al., 2012).

Other cytoplasmic targets of miRNAs that play a role in the male germline have also been proposed. Members of the miR-17-92 cluster are highly expressed in undifferentiated spermatogonia, where they are thought to play a role in promoting proliferation and inhibiting differentiation (Tong et al., 2012). The stimulation of spermatogonia to differentiate leads to a decrease in miR-17-92 cluster expression and an increase in transcript levels of *Bcl2l11*, *Kit* and *Socs3* (Fig. 3), three predicted targets of miR-17-92 with important roles in spermatogonial apoptosis and differentiation (Tong et al., 2012). In addition, one member of the cluster, the miR-18 family, is suggested to play a role during later stages of spermatogenesis. The miR-18 family is preferentially expressed in testis, and its levels peak during meiosis (Björk et al., 2010). Among its targets are mRNAs that encode the DNA damage repair protein ATM (Modzelewski et al., 2015), and a protein that plays a role in spermatid chromatin condensation, HSF2 (Björk et al., 2010). Both *Atm* and *Hsf2* transcript levels show an inverse correlation with miR-18 levels during spermatogenesis (Björk et al., 2010; Modzelewski et al., 2015). There are numerous other examples of potentially interesting miRNA-target relationships in the mammalian testis (McIver et al., 2012). However, because each miRNA regulates multiple mRNA targets, it is difficult to identify and decipher which regulatory interactions are causative of a phenotype when disrupted. Therefore, to prove that regulation of a particular mRNA by a specific miRNA contributes to normal germline function, it will be necessary to generate mice in which the target site(s) for that miRNA alone are disrupted within a particular gene. Indeed, with the growing power of clustered regularly interspaced short palindromic repeats (CRISPR) technology, such experiments are likely to become the standard in miRNA target studies.

### Roles in heterochromatin formation and transcriptional silencing

During meiosis, unpaired DNA is silenced (Turner, 2015), and this includes silencing of the X and Y chromosomes, which occurs via a specialized process known as meiotic sex chromosome inactivation (MSCI) and results in the compartmentalization of sex chromosomes into a specialized subdomain known as the sex body. After completion of meiosis, a period of elevated transcriptional activity follows, after which all gene expression is silenced and DNA is tightly packaged onto protamines during spermatid elongation (Braun, 1998). Heterochromatin formation and transcriptional silencing, therefore, are crucial at multiple stages of spermatogenesis. There is now accumulating evidence to suggest that AGO proteins can play a role in these events, particularly during meiotic silencing. For example, mice lacking AGO4, which localizes to the nucleus during meiosis in males, are subfertile and exhibit defects in meiotic silencing of the X and Y chromosomes (Modzelewski et al., 2012). In addition, fluorescence *in situ* hybridization experiments have suggested that miRNAs display distinct nuclear localization patterns, particularly surrounding the transcriptionally silenced X and Y chromosomes and, in some instances, the autosome cores (Marcon et al., 2008). Moreover, 11 of the 28 miRNAs preferentially or specifically expressed in the testis are found on the X chromosome (Ro et al., 2007), suggesting that the X chromosome is enriched for small RNAs expressed during spermatogenesis. Although one recent study has suggested that X chromosome-encoded miRNAs are also subject to MSCI, and demonstrated that improper expression of certain miRNAs during pachytene leads to meiotic defects (Royo et al., 2015), alternative studies have suggested that miRNAs on the X chromosome escape MSCI (Song et al., 2009; Sosa et al., 2015). Notably, the loci corresponding to a subset of X chromosome-encoded miRNAs localize to the periphery of the sex body during MSCI and escape silencing, continuing to be expressed during pachytene (Sosa et al., 2015). Given that defects in sex body formation are found in mice lacking AGO4 (Modzelewski et al., 2012), together with the unique spatial patterning of transcriptionally active miRNA loci in the sex body (Sosa et al., 2015), one possibility is that AGO-bound miRNAs play a role in the structural organization of the sex body. If miRNA precursors are continually transcribed during pachytene, miRNAs processed from these precursors could be loaded onto AGO proteins that find their way back into the nucleus, now able to target their own nascent precursor transcripts and recruit proteins important for the spatial organization of the sex body to those sites (Fig. 3). Characterization of the small RNAs, proteins, and chromatin regions that nuclear AGOs interact with in the male germline, along with the temporal order of these interactions relative to establishment of the sex body, will be crucial steps towards determining the roles these X chromosome-encoded miRNAs play during spermatogenesis.

A role for mammalian AGO-proteins and their associated RNAs in heterochromatin formation and transcriptional silencing is not unexpected. In lower eukaryotes, small RNAs contribute to equivalent silencing processes through direct interaction with nascent transcripts in the nucleus. In *S. pombe*, for example, *Dicer*-dependent small RNAs produced from sense and antisense centromeric transcripts guide a protein complex to centromeric chromatin via base-pairing with nascent transcripts (Reinhart and Bartel, 2002; Verdel et al., 2004; Volpe et al., 2002). This centromere silencing complex, termed the RNA-induced transcriptional silencing (RITS) complex, includes both the chromodomain protein Chp1 and the sole *S. pombe* AGO protein, and is necessary for heterochromatin formation and transcriptional silencing of the region (Bühler et al., 2006; Verdel et al., 2004). Similar involvement of nuclear AGO-bound small RNAs in heterochromatin formation is observed in the fly soma (Fagegaltier et al., 2009). Furthermore, disruption of the *Dicer*-like gene (Alexander et al., 2008) or the sole *Ago* gene in the fungus *Neurospora crassa* (Lee et al., 2003) prevents silencing of unpaired DNA during meiosis; both of these genes are also required for fertility (Aramayo and Selker, 2013). In *C. elegans*, HRDE-1, a member of the WAGO (for worm-specific AGO) clade of Argonautes, acts in the germline to guide small RNAs into the nucleus to target nascent pre-mRNAs and recruit transcriptional silencing co-factors (Ashe et al., 2012; Buckley et al., 2012; Grishok, 2013). Loss of HRDE-1 leads to an accumulation of silencing defects over multiple generations, ultimately resulting in sterility (Buckley et al., 2012). Interestingly, it has been proposed that the HRDE-1 pathway acts with the piRNA pathway to silence unpaired DNA; the piRNA pathway initially acts to detect and silence unpaired DNA whereas the HRDE-1 pathway maintains silencing over multiple generations (Ashe et al., 2012). Finally, using cultured human cells, AGO1, AGO2 and DICER were shown to contribute to establishment of the repressive H3K9me2 mark that appears at R-loop-forming terminator regions, which promote Pol II pausing at these regions and efficient termination of transcription (Skourti-Stathaki et al., 2015). AGO2 was also shown to interact with the SWI/SNF chromatin-remodeling complex in cultured mammalian cells, with DICER-dependent small RNAs mapping to SWI/SNF-bound transcriptional start sites (Carissimi et al., 2015). These data, taken together, suggest that AGO-bound small RNAs can directly influence the state of chromatin in other model systems and also in the mammalian nucleus, at least in cell culture experiments.

In summary, there is increasing evidence that AGO-bound small RNAs influence chromatin organization within the mammalian nucleus. Moreover, such nuclear roles also encompass the male germline, where AGO-bound small RNAs appear to play a role in sex body formation during meiosis.

### Roles in DNA damage repair

A number of studies have also revealed the potential involvement of AGO proteins and AGO-associated RNAs in the DNA damage response (DDR) pathway (Francia et al., 2012; Gao et al., 2014; Michalik et al., 2012; Wei et al., 2012). For instance, experiments in cultured human cell lines have identified small RNAs and components of the small RNA machinery that contribute to DNA damage repair (Francia et al., 2012). Part of the response involves the formation of DDR foci, which are aggregates of repair proteins that form at sites of double-strand breaks (Rothkamm et al., 2015); DROSHA, DICER and AGO2 were recently reported to contribute to the formation of DDR foci in irradiated somatic mammalian cells (Francia et al., 2012). Crucially, the knockdown of *TNRC6A*, *B* and *C*, homologs that are collectively essential factors in the canonical miRNA post-transcriptional regulatory pathway, had no impact on DDR foci (Francia et al., 2012). These data suggest that AGO-bound small RNAs do not simply indirectly affect DDR foci formation through the post-transcriptional regulation of classical DDR factors or pathways, but instead function via a novel mechanism. Furthermore, induction of a double-strand break (DSB) at a defined locus results in the generation of locus-specific small RNAs, presumably acting in *cis*, and these DROSHA- and DICER-dependent small RNAs are necessary for maximum DDR foci formation (Francia et al., 2012). A similar phenomenon has been observed in plants (Wei et al., 2012) and flies (Michalik et al., 2012). Furthermore, in mammals, interactions between nuclear AGO2 and the DNA damage repair protein RAD51 have been reported, with AGO2 being essential for the recruitment of RAD51 to DSBs (Gao et al., 2014). Although the identity of DNA damage-associated small RNAs remains poorly characterized, studies suggest that they cannot be miRNAs acting in *cis*, as the number and distribution of possible DSB loci far exceeds the number of miRNA-encoding loci. Thus, these small RNAs are either *Drosha*-dependent siRNAs or a novel class of small RNA. How these small RNAs are generated from DSB loci and how they promote accumulation of DNA repair complex formation remains unclear.

Given the crucial role that DSB repair plays in homologous recombination in germ cells, it is intriguing to consider whether non-canonical regulation of DDR foci by small RNAs occurs during mammalian meiosis. As male mammalian germ cells enter prophase I of meiosis, the protein SPO11 creates multiple DSBs across the chromosomes, activating DNA damage repair at those sites via homologous recombination (Keeney et al., 1997; Lam and Keeney, 2015). The only connection found, to date, between DNA damage repair and small RNAs in male germ cells was the observation of chromosomal instability in *Dicer*- and *Dgcr8*-deficient mouse spermatocytes, which results from fusions of the X and Y chromosomes to autosomes and one another (Modzelewski et al., 2015). However, multiple DNA damage repair proteins are also dysregulated in *Dicer*- and *Dgcr8*-deficient spermatocytes, including ATM, a key regulator of the repair response, which is regulated by multiple germline miRNAs. Thus, chromosomal instability in miRNA-deficient germlines may simply derive from a loss of canonical post-transcriptional regulation of DSB repair machinery. To test whether small RNAs directly participate in DNA damage repair during male meiosis (Fig. 3), it will be crucial to determine whether small RNAs are derived from DSB loci in the germline. Numerous methods exist for mapping the location of meiotic recombination hotspots (Hwang and Hunter, 2011; Smagulova et al., 2011), and this information, combined with small RNA sequencing data, could provide insights into roles for small RNAs in DSB repair.

## AGO-bound small RNAs carry out sexually dimorphic functions in gametogenesis

Although mammalian male and female gametes differ from one another in their morphology and cellular composition, both are generated from the highly regulated differentiation of PGCs into cells that undergo meiosis to produce gametes. Thus, perhaps small RNA-mediated gene regulation would be expected to be similar between males and females, especially before the first meiotic division. Instead, there is considerable sexual dimorphism in the requirement for small RNAs in gametogenesis (Table 1). Furthermore, the nature of this dimorphism varies among mammals.

**Table 1.**
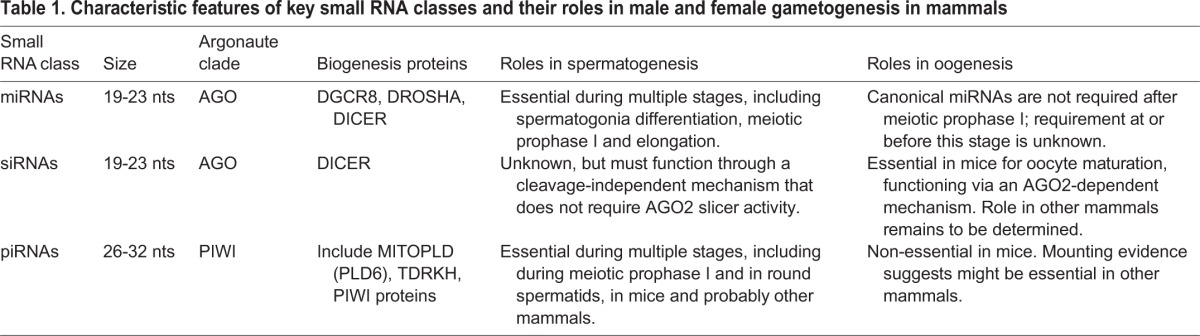
**Characteristic features of key small RNA classes and their roles in male and female gametogenesis in mammals**

**Fig. 4.**
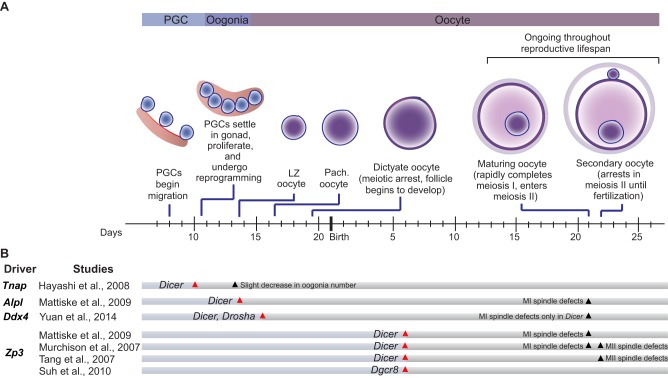
**Oogenesis in mice and the effects of *Dgcr8*, *Drosha* and *Dicer* female germline knockout.** (A) In female mice, PGCs begin to migrate to the gonad at E8. By E10.5, they settle in the gonad and begin a short program of proliferation and reprogramming, becoming oogonia. Soon after, at around E13.5, all oogonia enter meiosis, becoming oocytes. Around the time of birth, oocytes arrest in the diplotene stage of meiotic prophase I and follicle development begins; this stage is known as dictyate arrest and lasts until puberty, when subsets of oocytes will be triggered by pituitary gonadotrophins to mature. The timing of the first wave of oocyte maturation varies, typically happening between P14 and P21. This maturation coincides with ovulation, and involves the completion of meiosis I and progression through meiosis II until metaphase, at which point the oocyte once again arrests. The mature oocyte can remain arrested for many days in metaphase II until fertilization. (B) The conditional knockout of genes encoding small RNA biogenesis factors (*Dgcr8*, *Drosha* or *Dicer*) reveals essential roles for AGO-bound small RNAs during oogenesis. Summarized here are studies in which *Dgcr8*, *Drosha* or *Dicer* were conditionally disrupted from the female mouse germline. The promoter driving Cre-mediated disruption in each case is indicated, together with the approximate time of Cre activation (red arrowhead) and the time point at which a phenotype was observed (black arrowhead). Timescale indicates embryonic days up until birth, then postnatal days. MI, meiosis I; MII, meiosis II (MII); LZ, leptotene/zygotene; Pach., pachytene.

Unlike in male mice, in which miRNAs appear to play important roles throughout spermatogenesis, miRNAs are dispensable for the later stages of oogenesis (Fig. 4), including oocyte maturation (Suh et al., 2010). Indeed, the miRNA pathway is downregulated during oogenesis (Ma et al., 2010; Suh et al., 2010). It is instead siRNAs that are the AGO-bound small RNA necessary for the later stages of oocyte development (Murchison et al., 2007; Stein et al., 2015; Suh et al., 2010; Tang et al., 2007). Why might this be? Whereas piRNAs and the piRNA machinery are abundant in the male germline, and play important roles in repressing mRNAs and transposable elements, they are far less abundant in mouse oocytes and are not required for oogenesis (Carmell et al., 2007; Deng and Lin, 2002; Kuramochi-Miyagawa et al., 2004). However, the repression of transposable elements still appears to be important for oogenesis, as a higher proportion of oocytes from mice expressing elevated levels of LINE-1 retrotransposons undergo apoptosis during meiotic prophase I (Malki et al., 2014). Instead, a subclass of siRNAs ensures repression of transposable elements in the female mouse germline (Stein et al., 2015; Watanabe et al., 2008). *Ago2* is also essential for proper mouse oocyte maturation (Kaneda et al., 2009); female germline siRNAs thus probably function via a target cleavage mechanism, which does not occur in the male germline, given the dispensability of *Ago2* for male gametogenesis. It is unknown why the miRNA pathway is downregulated in murine oocytes. One explanation is that in females, transposon-silencing siRNAs would have to compete with miRNAs for available AGO proteins, whereas in males the usage of AGO-independent small RNAs, namely piRNAs, avoids such competition. Together, these observations have led to the dogma that in all mammals, siRNAs are the required class of small RNA for oogenesis, and miRNAs and piRNAs the required small RNAs for spermatogenesis.

Growing evidence, however, suggests this paradigm, with only siRNAs required for oogenesis, might be the exception rather than the norm in mammals. In mice, a retrotransposon that becomes activated in oocytes is found within the *Dicer* gene, leading to the expression of a unique, truncated isoform of DICER in mouse oocytes that more effectively generates siRNAs from dsRNAs (Flemr et al., 2013). This might explain why in oocytes, siRNAs are effectively generated from dsRNAs, whereas in mouse somatic cells, which do not express the truncated isoform of DICER, siRNAs are not produced effectively (Flemr et al., 2013). However, this insertion event has occurred relatively recently, and is only found in the *Muridae* family, which includes mice and rats. Therefore, if siRNAs play an important role in the repression of transposons in mouse oocytes, but are only effectively produced by a form of *Dicer* unique to the *Muridae* family, how are the deleterious effects of transposons kept at bay in other mammals that lack this unique *Dicer* isoform? It has recently been revealed that PIWI proteins and piRNAs are dynamically expressed in human, macaque and bovine ovaries, suggesting that piRNAs could repress transposons in oocytes (Roovers et al., 2015). Mice (and rats) are the exception in another regard: unlike most eutherian mammals, which have four copies of PIWI proteins (PIWIL1, 2, 3 and 4), they have lost PIWIL3. Notably, in bovine studies, PIWIL3 is found only in oocytes and is undetectable in testis (Roovers et al., 2015), suggesting that this PIWI paralog might play a specific role in cow oocytes, and perhaps also in the oocytes of other mammals. The concurrence of loss of PIWIL3 and the gain of a unique, oocyte-expressed *Dicer* isoform in the *Muridae* family has kindled speculation that in mice and rats AGO-bound siRNAs have supplanted a role typically performed by PIWIL3-bound piRNAs in other eutherian oocytes (Roovers et al., 2015). Disruption of *Piwil3*, as well as other PIWI genes, in mammalian organisms other than mice would determine whether piRNA pathways play essential roles in oogenesis in non-*Muridae* mammals.

The discrepancies among mammals regarding which classes of small RNAs are required for female gametogenesis highlight the evolutionary plasticity of germline biology. Furthermore, such discrepancies caution against building a paradigm of mammalian germline regulation based on observations from a single model organism. The apparent diversity in AGO-bound small RNA regulatory strategies in females prompts the question of whether those in males are similarly diverse. It is unclear whether siRNAs are essential for male gametogenesis in mice, and even less is known about their role in other mammals. As for miRNAs, the miR-34 family is highly expressed in the mature testis of many other mammals, including pig, rhesus monkey and cow (Lian et al., 2012; Tscherner et al., 2014; Yan et al., 2009), and its loss is correlated with infertility in humans (Abu-Halima et al., 2013, 2014; Wang et al., 2011). Thus, functions for miR-34 are likely to be conserved across mammals. It should be noted that, whereas the sexually dimorphic nature of AGO-bound small RNA regulation has been explored during the later stages of gametogenesis in mice, small RNA studies during earlier stages of oogenesis are lacking. Thus, although miRNAs are often assumed to be dispensable for the entirety of oogenesis, the only studies directly investigating the requirement for miRNAs in oogenesis used an approach involving conditional disruption of miRNA biogenesis during prophase I (Fig. 4), and therefore could only investigate roles for miRNAs at later stages of oocyte development (Suh et al., 2010; Yuan et al., 2014). In addition, although two studies have attempted to remove *Dicer* at early stages of oogenesis and reported no major defects prior to oocyte maturation, approximately half of the germ cells at these earlier stages failed to undergo *Dicer* excision (Hayashi et al., 2008; Mattiske et al., 2009). As miRNAs are important in males for both early germ cell differentiation and progression through the pachytene stage of meiotic prophase I, indicating requirements at or before both of these stages, further studies of AGO-bound small RNAs in earlier stages of oogenesis will reveal whether the requirement for miRNAs early on in gametogenesis is, in fact, shared between males and females.

## Conclusions

The studies reviewed here reveal that AGO proteins and AGO-bound RNAs are increasingly implicated in many of the events occurring during spermatogenesis, although it is clear that further studies are needed to fully define their functions in this context. Notably, of the thousands of AGO-bound small RNAs identified in the male germline, only the miR-34 family has been definitively shown to be essential for spermatogenesis. Thus, roles for the majority of small RNAs in the germline remain to be determined. Whether the infertility of *Dgcr8* and *Drosha* germline cKO mice is caused by the loss of miRNA families in addition to miR-34 will be most efficiently answered by the generation of additional miR-family knockouts, with highly expressed miRNAs demonstrating unique germline expression patterns or those correlating with infertility being the most promising candidates. Such candidates include members of the miR-17-92 cluster, which appear to play a role in the early stages of spermatogenesis, during spermatogonia differentiation. As illustrated by early attempts to study the miR-34 family, redundantly functioning miRNAs are encoded in multiple genomic locations, necessitating a complex knockout strategy. The advent of the CRISPR system greatly simplifies the process of creating multi-loci knockouts, allowing for comprehensive disruptions of miRNA families in order to study their role in spermatogenesis. Given the essential roles many miRNAs play in other tissues, cKO strategies will also be needed in cases where whole-animal knockouts are lethal.

Perhaps the major outstanding question relating to mammalian germline small RNAs regards the existence of their roles beyond post-transcriptional gene regulation. Given that AGO proteins are present in the nucleus of male germ cells, and that examples of nuclear roles for AGO proteins in other organisms or other cell types are plentiful, it seems probable that they function in a manner beyond post-transcriptional gene regulation during spermatogenesis. Why then has so little robust evidence for nuclear AGO function in the male germline been found? One possibility is that such alternative functions do exist, but that the dramatic phenotypes that result from loss of miRNA regulation of mRNAs in *Dicer* cKOs mask the phenotypes that are due to nuclear AGO function. In addition, the inability to maintain male germ cells in culture (Handel et al., 2014) or to easily purify them from surrounding somatic cells has greatly hindered their characterization at specific developmental stages. However, the recently described successful *in vitro* differentiation of ESCs into functional spermatozoa (Zhou et al., 2016) overcomes one of these barriers, giving researchers the ability to study meiosis in cultured cells. The development of technologies that could efficiently purify large numbers of germ cells would also make the biochemical investigation of nuclear AGOs and their small RNAs in germ cells much easier. Another caveat is that current methods provide average expression levels of small RNAs from a pool of cells when, in fact, levels of certain small RNAs might vary drastically within apparently homogeneous cell populations (Rissland et al., 2011). The development of single-cell small RNA sequencing will provide a more accurate depiction of miRNA profiles throughout spermatogenesis. It is also possible that, although AGOs might be present in the nucleus, they may no longer play important roles there; perhaps piRNAs have evolved in male germ cells to possess most or all of these alternative functions, leaving AGOs to focus solely on the task of miRNA-mediated post-transcriptional regulation.

Beyond the identities and roles of AGO-bound small RNAs lies a more complex question, which the male germline – home to all three classes of small RNAs – is uniquely poised to answer: how do the functions and pathways mediated by the three classes of small RNAs interconnect? If siRNAs do play a role in the germline, might they interact with the piRNA pathway in a manner similar to WAGO-associated small RNAs in *C. elegans*, which ensure long-term silencing of transposable elements? Could interactions exist between the miRNA and piRNA pathways? Answering these questions will help to explain how male mammalian germ cells are able to coordinate the dramatic chromatin rearrangements, genetic reprogramming and cellular morphogenesis that drive spermatogenesis. The answers to these questions are also likely to shed light on events occurring in somatic cells.
